# Adaptation and Validation of the Compassionate Capacity Scale for Portuguese Healthcare Students (CCS-PHS)

**DOI:** 10.3390/bs15081039

**Published:** 2025-07-31

**Authors:** María Dolores Ruiz-Fernández, Andrea Alcaraz-Córdoba, Irma Brito, Maria Jacinta Dantas, Tania Alcaraz-Córdoba, Angela María Ortega-Galán

**Affiliations:** 1 Department of Nursing, Physiotherapy and Medicine, Faculty of Health Sciences, University of Almería, 04120 Almería, Spain; mrf757@ual.es (M.D.R.-F.); tac943@ual.es (T.A.-C.); 2 Facultad de Ciencias de la Salud, Universidad Autónoma de Chile, Providencia 4780000, Chile; 3 Torrecardenas University Hospital, Andalusian Health Service, 04009 Almería, Spain; 4 Executive Committee of International Collaboration for Participatory Health Research (ICPHR), 3045 Coimbra, Portugal; irmabrito@esenfc.pt; 5 Nursing School of Coimbra & UICISA:E, 3004-011 Coimbra, Portugal; jacintadantas@esenfc.pt; 6 Almería Health District, Andalusian Health Service, 04009 Almería, Spain; 7 Department of Nursing, Physiotherapy and Medicine, University of Huelva, 21071 Huelva, Spain; angela.ortega@denf.uhu.es

**Keywords:** compassion capacity, compassion, healthcare students, instrument validation, scale

## Abstract

Compassion is a critical competence for healthcare students. However, there are no tools that measure compassionate capacity during students’ training. Recently, the Compassion Capacity Scale (CCS) was developed for health professionals, exhibiting good psychometric properties. The aim of this study was to translate, culturally adapt, and validate the CCS for Portuguese healthcare students. The study was divided into two phases: (1) translation and adaptation of the CCS for Portuguese students in the healthcare field; (2) validation and analysis of psychometric properties. The CCS-PHS showed good internal consistency (Cronbach’s α = 0.886), temporal stability (rho = 0.703), and content validity (CVI-i = 1). Criterion validity analysis showed strong correlations between all of the CCS-PHS’s dimensions and the chosen reference criteria scale. Construct validity analysis revealed that the CCS-PHS is composed of 17 items, classified into four factors. The differences found in the exploratory factor analysis in relation to the original scale may be due to the life experiences of healthcare professionals when compared to those of students. Our psychometric analysis suggests that the CCS-PHS is a reliable and valid tool to assess compassionate capacity in healthcare students. Knowing the compassionate competence of students is vital for guiding educational strategies, implementing compassion training programs and evaluating their effectiveness, as well as reinforcing key attitudes and behaviors for humanized and ethical healthcare.

## 1. Introduction

Young adults, especially university students, have high levels of stress and other mental disorders such as anxiety or depression (Tendhar & Bueno de Mesquita, 2020), and the mental health of this population is considered to be of global concern (Callow et al., 2021; Martínez-Rubio et al., 2021). Specifically, higher levels of perceived stress have been identified in healthcare students in the healthcare field (Alwhaibi et al., 2023). This is related to the fact that training encompasses the clinical and academic environments (Felicilda-Reynaldo et al., 2017) and involves clinical practice in highly sensitive environments with little margin for error (Lepiani-Díaz et al., 2023). The presence of high levels of stress may pose a risk to trainees’ well-being (Alquwez et al., 2021; Poots & Cassidy, 2020), negatively affecting the learning process and the delivery of compassionate care (Alabdulaziz et al., 2020). Compassion is defined as sensitivity to one’s own and others’ suffering, with a commitment to try to alleviate and prevent it (Gilbert, 2014). In recent years, compassion has gained increasing importance in healthcare (Sinclair et al., 2016a, 2016b) due to personal benefits such as improvements in mental and physiological health, emotional regulation, or interpersonal and professional relationships (Matos et al., 2022), highlighting a positive impact on patient experience and quality of care indices (Sinclair et al., 2021a). Although compassionate care is recognized as a standard of care and an essential component of healthcare (Malenfant et al., 2022), the literature suggests that its practice is deficient, highlighting the need for healthcare students to develop this skill throughout their training (Edoho Samson-Akpan et al., 2022; Sinclair et al., 2021b). Traditional training tends to focus on the acquisition of theoretical knowledge (Menezes et al., 2021; Sinclair et al., 2016b), underestimating the importance of human aspects such as compassion, empathy, and/or interpersonal skills (Sinclair et al., 2021b). This orientation has contributed to the dehumanization of healthcare and the consolidation of a value system focused on technical skills, speed, and efficiency (Głębocka, 2019).

In this context, various studies suggest that healthcare students may present compassion as an intrinsic quality, which may be preserved and strengthened through appropriate educational interventions within the framework of clinical training and personal experiences (Santiago et al., 2022; Zarrinkolah et al., 2025), thus consolidating it as an integral component of the training process (Rashedi et al., 2015). However, there is no specific undergraduate training to develop or cultivate compassion (Sinclair et al., 2016b), despite it being considered a critical competence of healthcare professionals (Sinclair et al., 2020), and the tools available for its measurement are limited (Gu et al., 2017; Strauss et al., 2016) and present variations depending on whether they target healthcare professionals or students (Sinclair et al., 2022). This suggests the need to develop methods to teach, simulate, and assess compassion (Hofmeyer et al., 2016).

The Capacity for Compassion Scale (CCS) has recently been developed, exhibiting good psychometric properties, and is the only scale available to measure compassionate capacity in healthcare professionals (Ruiz-Fernández et al., 2023). The scale has 17 items, organized into four dimensions: ‘self-compassion’, ‘shared humanity’, ‘motivation/engagement’, and ‘presence’. For university students, only the Bolton Compassion Strengths Indicators scale (Durkin et al., 2020) is available to measure indicators of compassion in nursing students. This scale was based on the theoretical Compassion Strengths Model (Durkin et al., 2019) and has 48 items, divided into eight dimensions: empathy, connection, self-care, communication, interpersonal skills, competence, engagement, and character. The CCS and Bolton Compassion Strengths Indicators measure different theoretical concepts. More specifically, the Bolton Compassion Strengths Indicators do not include dimensions such as shared humanity, self-compassion, or presence. Shared humanity refers to the awareness that suffering is a common experience, facilitating empathic connection with others (Uluğ et al., 2022). On the other hand, self-compassion contributes to the emotional well-being of professionals and prevents burnout or compassion fatigue (K. D. Neff & Germer, 2013). A number of studies identify that psychometric instruments designed to assess compassion should incorporate both shared humanity and self-compassion, considering that these critical subdimensions are able to measure the construct of compassion (K. Neff, 2003; K. D. Neff, 2023; Pommier et al., 2020; Strauss et al., 2016). In this regard, Strauss et al. (2016) noted that many existing compassion scales lack essential components and identified the need to adapt or develop more theoretically grounded instruments. In addition, the literature suggests that questionnaires with numerous variables increase fatigue, boredom, and exhaustion of the participants and may affect the quality of the data collected (Mourão et al., 2022). Considering that the Bolton Compassion Strengths Indicators has a larger number of items than the CCS, another positive aspect of the CCS is the small number of items. Furthermore, the need for linguistic and cultural adaptations of psychometric scales with a rigorous methodology to ensure their validity in new contexts is described (Beaton et al., 2000; Guillemin et al., 1993), since literal translation can generate semantic and conceptual equivalence failures, affecting the reliability and validity of the instrument (Guillemin et al., 1993; Ramada-Rodilla et al., 2013). Specifically, different studies conclude that many compassion tools lack adaptations, which compromises their applicability and accuracy in culturally diverse contexts (Jiang et al., 2023; Sinclair et al., 2017, 2022).

For the above reasons, the need to culturally adapt and validate the CCS for use by healthcare students is justified. This adaptation not only ensures a more accurate measurement of compassion but also helps to reinforce the validity of the instrument and its practical applicability in the training processes of health professions. Therefore, the aim of this study was to translate, culturally adapt, and validate the Compassionate Capacity Scale for Portuguese Healthcare Students (CCS-PHS).

## 2. Materials and Methods

### 2.1. Design

A two-phase study was conducted for the adaptation and psychometric validation of the CCS-PHS. Prior permission was sought from the Ethics Committee to carry out the research.

#### 2.1.1. Phase 1: Translation and Adaptation of the CCS for Healthcare Students

*A.*  *Protocol for Translation–Back Translation of the CCS*

For the adaptation of the CCS questionnaire developed for healthcare professionals for students in the healthcare field, a translation–back translation of the scale was first performed, following the steps described by Beaton et al. (2007). The translation–back translation protocol consisted of translation into Portuguese independently by two native Portuguese translators. After completing the translation, the two translators discussed the two versions, resolving inconsistencies and reaching consensus on the first translated version of the scale. This version was then back-translated by two native Spanish translators who were fluent in Portuguese, thereby obtaining two back-translated versions that were unified by checking their equivalence with the original Spanish version. The next phase consisted of the analysis of the Portuguese version by a committee of experts for semantic, idiomatic, experimental, and conceptual equivalence, which is described further in Section B.

*B.*  *Delphi Panel’s analysis of the CCS-PHS*

A group of university health students were selected and voluntarily agreed to be part of the expert committee in order to adapt the CCS to the context of the students.

The expert committee reflected on the key elements for assessing compassionate capacity in university students, applying the Delphi Method in order to reach a consensus. The Delphi Method is a process of refining survey items based on two or more phases. In each phase, participants will be asked how they rate each item and reflect on the relevance of the survey items to a chosen topic (Beiderbeck et al., 2021). The Delphi Panel in our study had two phases. In the first phase, students reflected on what compassionate capacity is through the following open-ended questions: ‘What is compassion?’, ‘What is self-compassion?’, ‘What is compassionate capacity?’, and ‘What is necessary to provide compassionate care?’. The responses to the questions were collected through the Mentimeter application. The Self-Compassion Scale (SCS) was then administered to improve understanding of the concepts. The respondents were next asked to analyze the CCS-PHS, obtained through the translation–back translation process described in Phase A, assessing the degree to which the items of this scale measured compassionate capacity. A Likert scale was used with the following scores: (1) strongly disagree, (2) disagree, (3) neither disagree nor agree, (4) agree, and (5) strongly agree. In addition, a comment box was included for each item at the end of the scale, allowing participants to explain their thoughts, include missing items, or add suggestions on how to improve the items. In the second phase, the researchers presented the results obtained in the previous phase with the participants’ responses, facilitating reflection on their own and other participants’ opinions and enabling participants to reposition their own assessment accordingly (Dragostinov et al., 2022). The results of each phase were shared anonymously, avoiding any bias that might result from the influence of the personality or status of other expert participants (Barrett & Heale, 2020).

The completion of Phases A and B resulted in a final version with a cross-cultural and contextual adaptation of the CCS for Portuguese healthcare students, initially developed for health professionals, hereafter referred to as the CCS-PHS. Some representative examples of items from the adapted CCS-PHS were as follows: “Sinto muita vontade de prevenir o sofrimento, aliviá-lo e/ou evitá-lo” (“I feel a strong desire to prevent, alleviate, and/or avoid suffering”), “Sinto a necessidade de ajudar os outros” (“I feel the need to help others”), and “Sinto-me com força e coragem para lidar com o sofrimento” (“I feel strong and courageous enough to deal with suffering”).

#### 2.1.2. Phase 2: Validation and Analysis of Psychometric Properties of the CCS-PHS

In Phase 2, a pilot study was carried out to validate the CCS-PHS, followed by a quantitative, descriptive, and cross-sectional study in order to assess the psychometric properties of CCS-PHS.

### 2.2. Participants

#### 2.2.1. Phase 1: Adaptation and Validation of CCS-PHS

The participants in the expert committee were selected by means of convenience sampling, with the following characteristics: [1] they had to be university students in the healthcare field (nursing, physiotherapy, medicine, etc.), and [2] they had to voluntarily agree to participate in the study. For the number of participants, the recommendations of Shang (2023) were taken into account, with a minimum of 8 to 23.

#### 2.2.2. Phase 2: Validation and Analysis of the Psychometric Properties of the CCS-PHS

As in Phase 1, convenience sampling with the same characteristics was used for the selection of participants for the pilot study and the final validation. All selected participants were enrolled at the University of Coimbra (Portugal). Health students on university exchange and those who had not completed an internship in a healthcare institution were excluded. The recommendations of Coaley (2014) and Norman and Streiner (2014) were taken into account, using a minimum of 50 participants for the pilot study, and a minimum of 10 participants per item for the final validation study.

### 2.3. Ethical Considerations

Participants were informed about the purpose of the study. It was explained that participation was voluntary and anonymous and that they could leave the study at any time and stop answering without any type of consequence, prejudice, or harm. The data obtained were kept confidential and processed in accordance with the Organic Law on the Protection of Personal Data and Guarantee of Digital Rights 3/2018 (Jefatura del Estado, 2018). The recommendations for participatory research were also applied (International Collaboration for Participatory Health Research (ICPHR), 2022). The study obtained authorization from the Ethics and Research Committee of UICISA:E No 1010_02_2024 and the University of Almería EFM 307/24.

### 2.4. Procedure and Data Analysis

#### 2.4.1. Phase 1: Adaptation and Validation of CCS-PHS

A content analysis of the data obtained using the Delphi Method was carried out using Wordle.net. Wordle.net is an online tool that generates word clouds of a text, emphasizing the words that appear most frequently. The data collected from the initial phase were analyzed by grouping similar items together, as well as through frequency distributions of the questionnaire responses. Following the recommendations of Barrios et al. (2021), a minimum of 75% agreement among the participants of the expert committee was considered an accepted average threshold of consensus. Then, the data were analyzed to identify convergence and changes in respondents’ judgements or opinions. To determine the level of collective opinion, descriptive and inferential statistics of item ratings were used to provide participants with information on the collected opinion of the expert committee. This allowed participants to know what their response was in relation to that of the group.

#### 2.4.2. Phase 2: Validation and Analysis of the Psychometric Properties of the CCS-PHS

##### Pilot Study

Prior to the pilot study, the content validity index (CVI-i) of the scale was calculated using the item ratings provided by the expert committee in the first phase. To assess internal consistency, Cronbach’s α, the corrected item–total correlation (C-ICT), and Cronbach’s α if the item was removed were calculated. Values above 0.7 for Cronbach’s α were considered acceptable (Oviedo & Campo-Arias, 2005), and we considered that items contributed to the internal consistency of the scale if the C-ICT was greater than 0.3 and the Cronbach’s α of the scale did not increase significantly after removing them (Coaley, 2014). To assess the temporal stability of the scale, Spearman’s rho was calculated between participants’ tests and retest responses. Participants were asked to respond to the scale again after a time interval of 1 to 3 months.

##### Final Validation Study

For the analysis of the psychometric properties of the final scale, reliability was analyzed using the methods described in the pilot phase (Coaley, 2014; Streiner & Kottner, 2014). In relation to construct validity, an exploratory factor analysis (EFA) and Barlett’s test of sphericity were used, with a value of *p* < 0.05 being considered significant (Coaley, 2014; Furr, 2014). The Kaiser–Meyer–Olkin (KMO) test was used to check sample adequacy, with a value above 0.7 being considered adequate. Criterion validation was performed through a correlation between the CCS-PHS and the validated scales in the Portuguese SCS (Castilho et al., 2015) and PROQOL (Hudnall Stamm, 2009). All data analyses were performed with IBM SPSS Statistics 24.

## 3. Results

The CCS-PHS showed good internal consistency, temporal stability, and content and criterion validity. The construct validity analysis revealed that the CCS-PHS is composed of 17 items, classified into four factors. The obtained results are detailed below.

### 3.1. Cross-Cultural Adaptation of the Questionnaire

The steps described by Beaton et al. (2007) were followed in the adaptation of the CCS for healthcare students. Next, the multidisciplinary committee of experts carried out an evaluation of the translated version of the scale, resolving discrepancies and developing a pre-final version that ensured comprehensibility and verified equivalence to the context of the students. For this purpose, the Delphi Panel was carried out, maintaining the original meaning of most of the items, as they were appropriate to the students’ context. However, some changes were made, until a consensus of the expert committee was reached. Examples of these changes are as follows: in the introductory sentence, the term “trabalho” (work) was changed to “ensino clínico/estágio” (clinical teaching/internship), and semantic improvements were made to Items 6 (changing the term “fugir” (fleeing) to “ausentar” (leaving)), 9 (including the word “como” (like)), and 11 (modifying the term “surge” (emerges) “sinto” (I feel)). Finally, in Item 13, the word “todos” (all) was removed from the sentence “Faço os possíveis …” (I do what is possible), as the expert committee thought that the meaning of this word is different for each person and that it is difficult to identify the maximum that we can do. The scale was then piloted.

#### Socio-Demographic Characteristics

The expert committee was made up of eight participants (seven nursing students and one medical student). Regarding socio-demographic characteristics, four students were enrolled in the fourth year, three in the third year, and one in the fifth year. In relation to the sex of the participants, seven were female, and one participant identified as other sex.

### 3.2. Pilot Phase: Analysis of Psychometric Properties

#### 3.2.1. Content Validity

The content validity analysis identified that the overall CVI-i of the CCS-PHS was 1, with all items scoring as very or fairly relevant for measuring compassionate capacity.

#### 3.2.2. Reliability

The Cronbach’s α for the pilot study was 0.930, identifying good internal consistency. In the C-ICT analysis, all items scored above 0.3 (Table 1). Considering temporal stability, calculated through the correlation between ‘TOTAL_TEST’ (mean of the scores obtained in the first response of the pilot sample) and ‘TOTAL_RETEST’ (mean of the scores obtained in the second response of the pilot sample) was rho = 0.703, with *p* = 0.000.

### 3.3. Final Phase: Analysis of Psychometric Properties

#### 3.3.1. Characteristics of the Participants

For the validation of this scale, there were 273 participants, 17.3% (*n* = 41) of whom were male and 82.7% female (*n* = 196). With regard to degree, nursing students (97.8%) and medical students (2.2%) were included. The majority of participants were third-year nursing students in the academic years 23/24 and 24/25 (94.9%). In addition, there were first- and second-year nursing students (2.9%) and fourth-, fifth-, and sixth-year medical students (2.2%). In relation to age, the mean was 21.77 (SD = 3.652), with a range of 20 to 50 years. Six responses were missing for this variable.

#### 3.3.2. Reliability

The internal consistency analyses of the CCS-PHS showed a Cronbach’s α of 0.886. In addition, the Cronbach’s α was analyzed if one item was removed, obtaining CCI-T > 0.3 for all items of the scale. Therefore, it was concluded that all items were adequate, and that removing one item did not improve the internal consistency of the questionnaire (Table 2).

#### 3.3.3. Construct Validity

The KMO test was 0.899, and Bartlett’s sphericity was significant (x^2^(136) = 1510.385; *p* = 0.000), which allowed us to conclude that there are significant correlations between the attributes and that the factor analysis was appropriate, revealing the presence of four dimensions (Table 3). Four factors presented eigenvalues equal to or greater than one, explaining 60.068% of the variance (Table 4).

#### 3.3.4. Criterion Validity

A statistically significant correlation was found between the ‘presence’ dimension of the CCS-PHS and the ‘mindfulness’ dimension of the SCS (rho = 0.224; *p* = 0.001), between the ‘self-compassion’ dimension of the CCS-PHS and the ‘self-kindness’ dimension of the SCS (rho = 0.487; *p* = 0.000), between the ‘motivation/engagement’ dimension of the CCS-PHS and the ‘compassion satisfaction’ dimension of the ProQOL (rho = 0.418; *p* = 0.000), and the ‘shared humanity’ dimension of the CCS-PHS and the same dimension of the SCS (rho = 0.225; *p* = 0.000), as shown in Table 5.

## 4. Discussion

The aim of this study was to translate, culturally adapt, and validate the CCS for Portuguese healthcare students. The literature shows the lack of measurement tools on compassion, as well as the lack of cross-cultural adaptations of the available scales. More specifically, the scale developed by Ruiz-Fernández et al. (2023) is the only tool available to measure compassionate capacity in healthcare professionals, and adaptation to the context of healthcare students is necessary. The availability of scales that make it possible to know an individual’s compassionate capacity is vital in order to direct educational strategies, as well as to implement and evaluate compassion cultivation or training programs. The development of this ability allows healthcare students to improve their emotional regulation skills, which could contribute to better mental health and professional performance, ultimately improving the humanization of healthcare (Rodriguez-Moreno et al., 2024; Sinclair et al., 2021b). In addition, we can highlight as practical implications benefits for patients, such as greater satisfaction with healthcare, reduced anxiety, or a decrease in symptoms (Baguley et al., 2020; Salvador Zaragozá et al., 2021).

The psychometric properties of the questionnaire were analyzed through reliability, content, criterion, and construct validity. Regarding reliability, the CCS-PHS obtained excellent reliability both in the pilot phase (Cronbach’s α 0.930) and in the final phase (Cronbach’s α 0.886), with similar results to the original CCS (Cronbach’s α 0.855) that was validated in healthcare professionals (Ruiz-Fernández et al., 2023). In relation to temporal stability, the result was adequate (rho = 0.703; *p* = 0.000), exceeding the minimum acceptable value (0.7) (Fukui et al., 2019; Lodder et al., 2022). Having a measure that shows consistent results over time is essential to ensure that any observed differences in scores within individuals or groups across different moments truly reflect meaningful and reproducible changes (Gritti et al., 2025).

Thus, the obtained results showed that the CCS-PHS is a reliable instrument for measuring and studying the compassionate capacity of Portuguese healthcare students, with stable results over time. In terms of criterion validity, statistically significant correlations were obtained in all comparisons between scales measuring similar concepts. Regarding terms of content validity, the expert committee considered all items of the original scale to be essential for measuring compassionate ability in students, making slight modifications to improve comprehension and adapt it to the student context. In addition, the total CVI-i of the scale was calculated, and a score of 1 was obtained.

With regard to construct validity, the exploratory factor analysis showed, like the original version, four dimensions, ‘motivation/engagement’, ‘self-compassion’, ‘shared humanity’, and ‘presence’, with certain differences in relation to the factor loading of the items. Firstly, Items 11 and 12 were identified with the dimension ‘motivation/engagement’ in our scale as opposed to the original version, which loaded them on the dimension of ‘shared humanity’. On the other hand, Items 13 and 14 were loaded on the dimension of ‘presence’ instead of the dimension of ‘motivation/engagement’ as in the original scale.

In order to theoretically justify the changes in factor loadings observed in Items 11 and 12 of the CCS-PHS, we can draw on several theories and models. University students in the healthcare field, when compared to professionals, are at an early stage of training, where the focus tends to be more on empathy and shared humanity (Jia-Ru et al., 2022) rather than on engagement (Wang & Yu, 2021; Zhang et al., 2023). Lack of clinical experience may make ‘motivation/engagement’ a less prominent dimension, as they have not yet had enough practice to internalize this aspect. This phenomenon is consistent with Benner’s theory that early-stage practitioners have fewer concrete experiences that lead them to develop concrete clinical outcome-oriented motivation (Benner, 1984). Also, according to Davis’s theory on the development of empathy, empathy skills, and recognition of shared suffering, these qualities tend to develop in the early stages of healthcare training (Davis, 1983). This could explain why items referring to ‘shared humanity’ have a higher loading in healthcare students (Davis, 1983; Zhao et al., 2022). Bandura’s Self-Efficacy Theory also offers a relevant explanation. Healthcare students, as they have less clinical experience, may not feel as confident or motivated about their ability to cope with clinical challenges. Instead, they focus on their ability to recognize the suffering of others, which contributes to the higher factor loadings of items related to shared humanity in this group (Bandura, 1997).

On the other hand, Items 13 and 14, which in the original version were loaded factorially on the ‘motivation/commitment’ dimension, were loaded in our scale on the ‘presence’ dimension. This can be explained by differences in the interpretation of presence in the training context. Presence for health students can be understood as the first step to developing engagement in their professional career, as well as a manifestation of compassion, without the full ability to apply these concepts in practice. Because of their lack of clinical experience, the act of being available to patients may be the closest expression of compassion that they can offer at the time. As they gain more experience and confidence, this ‘presence’ may evolve into a more active and motivated engagement in their role as caregivers (Benner, 1984). Also, in recent years, a downward trend has been observed in the professional commitment of the new generations of healthcare students, particularly in Generation Y [millennials] and Generation Z (Tan & Chin, 2023). Younger generations tend to prioritize a work–life balance, which is reflected in a lower willingness to fully commit to a demanding career such as healthcare (Kim et al., 2024), in contrast to the values of previous generations, such as the Baby Boomers, who placed work at the center of their lives (Slepian et al., 2024). This emphasis on work–life balance has been linked to lower professional motivation and commitment, as observed in several studies (Tan & Chin, 2023; Zhao et al., 2022).

Considering the limitations of the study, convenience sampling was used, limiting the generalizability of the results. On the other hand, the majority of participants were nursing students due to the ease of access to the researchers’ sample. However, the recommendations of Coaley (2014) and Norman and Streiner (2014) regarding the minimum number of participants in the pilot and final phases to analyze the psychometric properties of an instrument were followed. In addition, the response interval for temporal stability varied between 2 and 6 weeks due to final exams and the beginning of seasonal holidays. The CCS-PHS was a self-administered tool, so a potential social desirability bias may be present. Furthermore, the use of EFA may also be considered a limitation. However, given that this was the first cultural adaptation of the instrument, EFA was appropriate for identifying latent factors without assuming the validity of the original model in a new linguistic and cultural context (Beaton et al., 2000; Boateng et al., 2018; Fabrigar & Wegener, 2012).

Future lines of research should consider validating the instrument in different populations of university students, as well as testing it in different languages and cultures.

## 5. Conclusions

The CCS-PHS demonstrated good psychometric properties in relation to reliability, temporal stability, and content, criterion, and construct validity. Our psychometric analysis suggests that the CCS-PHS is a reliable and valid tool for assessing compassionate capacity in healthcare students. Compassionate capacity is a critical competence that healthcare students should acquire or improve during their clinical training in order to avoid a technical skill-centered and dehumanized system. Therefore, knowing the compassionate competence of students is vital for guiding educational strategies, implementing compassion training programs, and evaluating their effectiveness, as well as reinforcing critical attitudes and behaviors for humanized and ethical healthcare. As this tool has proven to be reliable in assessing compassionate capacity, it is recommended to use this self-administered scale during the teaching–learning process of health professions in order to raise awareness of personal development needs in the face of the demands of the future profession.

## Figures and Tables

**Table 1:** Reliability of the pilot phase.

Item	Scale Average If the Item Has Been Deleted	Scale Variance If the Item Has Been Deleted	Corrected Correlation of Total of Items	Cronbach’s Alpha If the Item Has Been Deleted
1	66.18	72.436	0.651	0.926
2	65.96	73.958	0.695	0.925
3	66.68	74.222	0.587	0.927
4	66.32	72.181	0.613	0.927
5	66.36	72.521	0.642	0.926
6	66.24	71.941	0.828	0.922
7	66.48	73.724	0.553	0.928
8	66.18	72.436	0.651	0.926
9	65.98	72.836	0.759	0.924
10	66.12	73.006	0.706	0.925
11	66.08	73.626	0.718	0.925
12	66.62	73.996	0.521	0.929
13	66.26	71.298	0.786	0.923
14	66.72	73.593	0.596	0.927
15	66.60	73.673	0.517	0.929
16	67.06	73.894	0.514	0.929
17	67.12	70.965	0.669	0.926

**Table 2:** Reliability of the final phase.

Item	Scale Average If the Item Has Been Deleted	Scale Variance If the Item Has Been Deleted	Corrected Correlation of Total of Items	Cronbach’s Alpha If the Item Has Been Deleted
1	65.34	51.030	0.420	0.884
2	65.08	51.905	0.552	0.880
3	65.97	50.737	0.486	0.882
4	65.64	50.097	0.560	0.879
5	65.72	49.916	0.554	0.879
6	65.53	51.267	0.487	0.882
7	65.70	50.372	0.510	0.881
8	65.24	50.794	0.539	0.880
9	65.06	51.848	0.548	0.880
10	65.41	49.107	0.622	0.877
11	65.30	49.670	0.653	0.876
12	65.84	49.590	0.505	0.881
13	65.33	49.730	0.636	0.876
14	65.88	49.938	0.572	0.879
15	66.01	50.119	0.475	0.882
16	66.45	49.969	0.456	0.884
17	66.38	49.703	0.512	0.881

**Table 3:** Factor analysis of 4 dimensions.

Item	Component 1	Component 2	Component 3	Component 4
Motivation/Engagement Dimension				
1	0.192	0.143	−0.017	**0.746**
2	0.407	0.123	0.062	**0.705**
3	0.062	0.193	0.455	**0.521**
Presence Dimension				
4	0.293	**0.484**	0.187	0.259
5	0.176	**0.565**	0.251	0.223
6	0.179	**0.707**	0.053	0.096
7	0.225	**0.729**	0.155	−0.075
13	0.373	**0.478**	0.087	0.448
14	0.157	**0.658**	0.183	0.242
Shared Humanity Dimension				
8	**0.667**	0.152	0.068	0.296
9	**0.661**	0.117	0.041	0.392
10	**0.728**	0.227	0.248	0.105
11	**0.627**	0.311	0.183	0.277
12	**0.705**	0.288	0.032	0.006
Self-Compassion Dimension				
15	−0.056	0.325	**0.704**	0.193
16	0.169	0.134	**0.848**	−0.048
17	0.224	0.103	**0.851**	0.030

Notes: Extraction method: principal component analysis. Rotation method: Varimax with Kaiser normalization. The rotation converged in 6 iterations.

**Table 4:** Total variance explained.

Total Variance Explained									
Component	Initial Eigenvalues			Extraction Sums of Charges Squared			Rotational Sums of Squared Loads		
	Total	% Variance	% Accumulated	Total	% Variance	% Accumulated	Total	% Variance	% Accumulated
1	6.262	36.836	36.836	6.262	36.836	36.836	2.951	17.361	17.361
2	1.833	10.78	47.615	1.833	10.78	47.615	2.722	16.01	33.371
3	1.116	6.564	54.179	1.116	6.564	54.179	2.419	14.23	47.601
4	1.003	5.899	60.078	1.003	5.889	60.068	2.085	12.467	60.068
5	0.823	4.841	64.919						
6	0.817	4.804	69.723						
7	0.736	4.33	74.054						
8	0.575	3.379	77.433						
9	0.54	3.176	80.609						
10	0.53	3.119	83.728						
11	0.515	3.027	86.756						
12	0.462	2.715	89.47						
13	0.415	2.442	91.912						
14	0.396	2.327	94.239						
15	0.385	2.267	96.506						
16	0.32	1.884	98.39						
17	0.274	1.61	100						

Note. Extraction method: principal component analysis.

**Table 5:** Criterion validity.

	CCS-PHS					
			Presence	Self-Compassion	Motivation/Engagement	Shared Humanity
SCS	Mindfulness	rho	0.224			
		*p*	0.001 *			
	Self-Kindness	rho		0.487		
		*p*		0.000 *		
	Shared Humanity	rho				0.225
		*p*				0.000 *
ProQOL	Compassion Satisfaction	rho			0.418	
		*p*			0.000 *	

Note: * *p* < 0.05.

## Data Availability

Data are available from the first author or corresponding author on reasonable request.
